# Modelling *Wolbachia* infection in a sex-structured mosquito population carrying West Nile virus

**DOI:** 10.1007/s00285-017-1096-7

**Published:** 2017-01-17

**Authors:** József Z. Farkas, Stephen A. Gourley, Rongsong Liu, Abdul-Aziz Yakubu

**Affiliations:** 1 Division of Computing Science and Mathematics, University of Stirling, Stirling, FK9 4LA UK; 2 Department of Mathematics, University of Surrey, Guildford, Surrey GU2 7XH UK; 3 Department of Mathematics, University of Wyoming, Laramie, WY 82071 USA; 4 Department of Zoology and Physiology, University of Wyoming, Laramie, WY 82071 USA; 5 Department of Mathematics, Howard University, Washington, DC 20059 USA

**Keywords:** *Wolbachia*, Sex-structure, West Nile virus, Epidemic, Stability, 92D30, 34D20, 34C11

## Abstract

*Wolbachia *is possibly the most studied reproductive parasite of arthropod species. It appears to be a promising candidate for biocontrol of some mosquito borne diseases. We begin by developing a sex-structured model for a *Wolbachia *infected mosquito population. Our model incorporates the key effects of *Wolbachia *infection including cytoplasmic incompatibility and male killing. We also allow the possibility of reduced reproductive output, incomplete maternal transmission, and different mortality rates for uninfected/infected male/female individuals. We study the existence and local stability of equilibria, including the biologically relevant and interesting boundary equilibria. For some biologically relevant parameter regimes there may be multiple coexistence steady states including, very importantly, a coexistence steady state in which *Wolbachia *infected individuals dominate. We also extend the model to incorporate West Nile virus (WNv) dynamics, using an SEI modelling approach. Recent evidence suggests that a particular strain of *Wolbachia *infection significantly reduces WNv replication in *Aedes aegypti*. We model this via increased time spent in the WNv-exposed compartment for *Wolbachia *infected female mosquitoes. A basic reproduction number $R_0$ is computed for the WNv infection. Our results suggest that, if the mosquito population consists mainly of *Wolbachia *infected individuals, WNv eradication is likely if WNv replication in *Wolbachia *infected individuals is sufficiently reduced.

## Introduction

*Wolbachia* is a maternally transmitted intracellular symbiont, and it is the most common reproductive parasite infecting a significant proportion of insect species, see e.g. O’Neill et al. (1997), Werren (1997). *Wolbachia* typically inhibits testes and ovaries of its host, and it is also present in its host’s eggs. It interferes with its host’s reproductive mechanism in a remarkable fashion. This allows *Wolbachia *to successfully establish itself in a number of arthropod species. Well-known effects of *Wolbachia* infections include cytoplasmic incompatibility (CI for short) and feminization of genetic males also known as male killing (MK for short), see e.g. Caspari and Watson (1959), Hoffmann and Turelli (1997), Telschow et al. (2005a, b). Another important well-known effect of *Wolbachia* infections is the inducement of parthenogenesis, see e.g. Engelstädter et al. (2004), Stouthamer (1997). All of these contribute to the fact that the mathematical modelling of *Wolbachia *infection dynamics is both interesting and challenging.

In recent decades a substantial number of mathematical modelling approaches have been applied to model different types of *Wolbachia *infections in a variety of arthropod species. Perhaps most frequently researchers have been focusing on the development of mathematical models for *Wolbachia *infections in mosquito species. Many of the earlier models took the form of discrete time matrix models, written for population frequencies, see e.g. Turelli (1994), Vautrin (2007), and the references therein. Using frequency-type models a number of researchers investigated the possibility of coexistence of multiple *Wolbachia *strains, each of which exhibits different types of the reproductive mechanisms mentioned earlier, see e.g. Engelstädter et al. (2004), Farkas and Hinow (2010), Keeling et al. (2003), Vautrin (2007). Among others, *Wolbachia *strains have been investigated as a potential biological control tool to eradicate mosquito borne diseases. Originally the focus has been on *Wolbachia *strains that induce life-shortening of their hosts. This is because for many vector borne diseases only older mosquitoes are of interest from the point of view of disease transmission. Therefore the use of (discrete) age-structured population models has become increasingly prevalent, see e.g. Rasgon and Scott (2004) and the references therein. Fairly recently, in McMeniman (2009) the results of laboratory experiments were reported envisaging a successful introduction of a life-shortening *Wolbachia *strain in the mosquito species *Aedes aegypti*. In McMeniman (2009) three key factors, namely, strong CI, low fitness cost and high maternal transmission rate, were identified as drivers of a successful introduction of the new *Wolbachia *strain into an *Aedes* population. To this end researchers have developed and analysed continuous age-structured population models for *Wolbachia *infection dynamics, which take the form of partial differential equations, see Farkas and Hinow (2010); which can often be recast as delay equations, see e.g. Hancock et al. (2011a, b).

In recent years there have been substantial modelling efforts to theoretically investigate the potential of biological control tools for limiting the impact of mosquito borne diseases. It is now widely recognised that biological control represents a viable alternative to established methods such as the use of insecticides and bed nets. Among others, the sterile insect technique has been investigated in the recent papers (Dufourd and Dumont 2012; Li 2011; Li and Yuan 2015). More recently, it was reported that particular strains of *Wolbachia *(completely or almost completely) block dengue virus replication inside the mosquito hosts, see for example (Blagrove 2012; Hoffmann 2011; Walker 2011). To this end Hughes and Britton (2013) developed a mathematical model for *Wolbachia *infection as a potential control tool for dengue fever. Their work suggests that *Wolbachia *may be effective as such a control measure in areas where the basic reproduction number $R_0$ is not too large. These recent results underpin the possibility that *Wolbachia *may be a promising candidate for biocontrol of mosquito borne diseases, in general. Besides dengue, West Nile virus (WNv) is another well-known mosquito borne disease of current interest. WNv infection cycles between mosquitoes (especially *Culex* species) and a number of species, particularly birds. Some infected birds develop high levels of virus in their bloodstream and mosquitoes can become infected by biting these infectious birds. After about a week, infected mosquitoes can transmit the virus to susceptible birds. Mosquitoes infected with West Nile virus also bite and infect people, horses, and other mammals. However, humans, horses, and other mammals are ‘dead end’ hosts. This virus was first isolated in the West Nile region of Uganda, and since then has spread rapidly, for example in North America during the past 12 years. Since there is no vaccine available, the emphasis has been mainly on controlling the vector mosquito species. Some recent experiments, see Hussain (2013), have confirmed that replication of the virus in orally fed mosquitoes was largely inhibited in the wMelPop strain of *Wolbachia*. Interestingly, in a recent paper, Dodson et al. (2014) demonstrated in laboratory experiments that the *w*AlbB *Wolbachia* strain in fact enhances WNv infection rates in the mosquito species *Culex transalis*. However, in Dodson et al. (2014) the *Wolbachia* was not a stable maternally inherited infection, but rather they infected transiently somatic mosquito tissues, and hence the *w*AlbB infection did not induce significant immune response in the mosquitoes. This is probably key to their findings. Here we will focus on modelling a maternally inherited *Wolbachia* infection in a population model, which hypothesizes a large number of successive generations. Nevertheless, the findings in Dodson et al. (2014) underpin the importance of *Wolbachia* research in general and highlight the importance of contrasting the findings of new theoretical, laboratory and field investigations.

In this work we introduce sex-structured models for *Wolbachia *infection dynamics in a mosquito population. This will allow us to incorporate and study the well-known effects of CI and MK of particular *Wolbachia *infections, simultaneously. First we will treat a model which only involves the mosquito population itself. Then we will use this model as a basis for a much more complex scenario incorporating WNv dynamics in a *Wolbachia *infected mosquito population. The full WNv model will naturally include the bird population, too.

## Model for a *Wolbachia* infected mosquito population without WNv

### Model derivation

We start by introducing a model for a *Wolbachia *infection in a sex-structured mosquito population, incorporating sex-structure using a well established approach originally due to Kendall (1949). More recent papers of Hadeler (2012) and Hadeler et al. (1988) derive and discuss sex-structured pair formation models in depth. We only model (explicitly) the adult population of mosquitoes. Our model allows us to take into account the well-known effects of cytoplasmic incompatibility (CI), incomplete maternal transmission, fertility cost of the *Wolbachia *infection to reproductive output, and male killing (MK), at the same time. We note that it was shown in Engelstädter et al. (2004) that a stable coexistence of MK and CI inducing *Wolbachia* strains is possible, in principle. Introduction of male killing *Wolbachia* strains in vector populations may have a significant effect on the disease dynamics, as typically only female mosquitoes are transmitting the disease. Also note that according to Walker (2011), those *Wolbachia *strains which cause greater disruption, as in the case of dengue transmission, confer greater fitness costs to the mosquitoes. This may well be the case for West Nile virus, hence we account for the reduced reproductive output in our model.

We deduce our starting model from basic principles. In particular, first we deduce mating rules arising at the individual level. Starting with the adult population of size *N*, we construct a random mating graph. This is a bipartite graph, not necessarily complete, in which each vertex has degree at most one. The vertices represent male and female individuals and edges represent realized matings. Let us denote by $M,M_w,F,F_w$ the numbers of un/infected males and females, respectively. For every adult mating pair, offspring is created according to the following rules. Below, *m*, *f* and $m_w,f_w$ denote uninfected/infected male/female individual, respectively. The parameter $\beta$ models the reduced reproductive output of *Wolbachia *infected females, $\tau$ measures maternal transmission in the sense that it is the probability that a *Wolbachia *infected mother passes on the infection to its offspring, *q* measures CI in the sense that when a *Wolbachia *infected male mates with an uninfected female, *q* is the probability that there is no viable offspring. Finally, $\gamma$ measures MK in the sense that it is the probability that a *Wolbachia *infected male larva dies during its development. A complete list of parameter values will be given later on. With this notation the mating rules are described below.

1. $m \times f$: create one pair of the same type (*m*, *f*).
2. $m \times f_w$: with probability $\beta$, create no offspring. This reflects the fecundity reduction due to the Wolbachia infection. In the complementary case, with probability $(1-\beta )\tau (1-\gamma )$, create a new pair $(m_w, f_w)$, at the same time with probability $(1-\beta )\tau \gamma$ create $(0,f_w)$, i.e. a female only brood. This accounts for male killing (MK). With probability $(1-\beta )(1-\tau )$ create a new pair (*m*, *f*).
3. $m_w\times f_w$ : same as above.
4. $m_w\times f$: with probability *q*, create no offspring. This is the effect of cytoplasmic incompatibility (CI). With probability $1-q$, create a new pair (*m*, *f*).

Notice that, in contrast to Farkas and Hinow (2010), the sex ratio at birth will not be 1 : 1, it is distorted due to male killing. Also we allow different mortality rates for males and females, in general. Therefore, even in the case when there is no male killing, the sex ratio would be distorted, in general. Also, we assume that any offspring resulting from CI crossing is uninfected.

We apply the mating rules described above to construct the birth function in our model. If the population sizes in the four compartments are denoted by $M,M_w,F,F_w$, respectively, then the total number of possible matings is $(M+M_w)(F+F_w)$. The total number of matings for example between uninfected males and infected females is $MF_w$. Hence the probability that a given mating of type $m\times f_w$ takes place is $\frac{MF_w}{(M+M_w)(F+F_w)}$. To compute the total number of matings per unit time we follow the harmonic mean birth function approach from Keyfitz (1972). Accordingly, the total number of matings is proportional to

2.1 $$\begin{aligned}&M\left( \frac{F+F_w}{M+M_w+F+F_w}\right) +M_w\left( \frac{F+F_w}{M+M_w+F+F_w}\right) \nonumber \\&\qquad +F\left( \frac{M+M_w}{M+M_w+F+F_w}\right) +F_w\left( \frac{M+M_w}{M+M_w+F+F_w}\right) \nonumber \\&\quad =2\frac{(M+M_w)(F+F_w)}{M+M_w+F+F_w}. \end{aligned}$$

Hence the birth rate of offspring arising for example from the mating between an uninfected male and an infected female is proportional to $\frac{MF_w}{M+M_w+F+F_w}$. We also naturally assume that there is competition between female individuals for finding an appropriate water reservoir to lay eggs. This is taken into account via a function $\lambda (F_{total})$ which we assume (at least in the first instance) to be a monotonically decreasing function of the total number of females $F_{total}$, to allow for this competition for nesting places. Though $\lambda (F_{total})$ is decreasing, it may approach a positive limit as $F_{total}\rightarrow \infty$. This is to allow for the fact that gravid females that cannot find a place to lay their eggs may destroy eggs previously laid by others, and lay theirs instead. Thus the overall egg-laying rate should approach a positive limit as $F_{total}\rightarrow \infty$, and therefore we assume that $\lambda (F_{total})$ is a decreasing function such that $\lambda (\infty )>0$. Based on the individual mating rules explained earlier, our model reads as follows.

2.2 $$\begin{aligned} M^{\prime }(t)&= - \mu _m M + \frac{\lambda (F_{total})}{N} (MF + (1-\beta )(1-\tau )(MF_w + M_wF_w)\nonumber \\&\quad + (1-q)M_wF), \end{aligned}$$

2.3 $$\begin{aligned} F^{\prime }(t)&= - \mu _f F + \frac{\lambda (F_{total})}{N} (MF + (1-\beta )(1-\tau )(MF_w + M_wF_w) \nonumber \\&\quad + (1-q)M_wF), \end{aligned}$$

2.4 $$\begin{aligned} M^{\prime }_w(t)&= - \mu _{mw} M_w + \frac{\lambda (F_{total})}{N} (1-\beta )\tau (1-\gamma )(MF_w + M_wF_w), \end{aligned}$$

2.5 $$\begin{aligned} F^{\prime }_w(t)&= - \mu _{fw} F_w + \frac{\lambda (F_{total})}{N} (1-\beta )\tau (MF_w + M_wF_w). \end{aligned}$$

A complete list of the variables, parameters and coefficient functions appearing in model (2.2)–(2.5) is given below.

- *M*: number of uninfected male mosquitoes.
- *F*: number of uninfected female mosquitoes.
- $M_w$: number of *Wolbachia *infected male mosquitoes.
- $F_w$: number of *Wolbachia *infected female mosquitoes.
- $M_{total} = M+M_w$, total number of male mosquitoes.
- $F_{total} = F + F_w$, total number of female mosquitoes.
- $N=M_{total}+F_{total}$, total number of mosquitoes.
- $\beta$: reduction in reproductive output of *Wolbachia *infected females.
- $\tau$: maternal transmission probability for *Wolbachia *infection.
- *q*: probability of cytoplasmic incompatibility (CI).
- $\gamma$: probability of male killing (MK) induced by *Wolbachia *infection.
- $\lambda (F_{total})$: average egg laying rate, which depends on the total number of female mosquitoes.
- $\mu _m$: per-capita mortality rate of uninfected male mosquitoes.
- $\mu _f$: per-capita mortality rate of uninfected female mosquitoes.
- $\mu _{mw}$: per-capita mortality rate of *Wolbachia *infected male mosquitoes.
- $\mu _{fw}$: per-capita mortality rate of *Wolbachia *infected female mosquitoes.

Model (2.2)–(2.5) is our starting point for a study of the *Wolbachia *infection dynamics in a sex-structured mosquito population. Later, we will expand this model by introducing WNv infection.

### Positivity and boundedness

First we begin by establishing positivity and boundedness of solutions of model (2.2)–(2.5).

#### Proposition 2.1

Assume that $\lambda$ is a monotone decreasing function such that

2.6 $$\begin{aligned} \displaystyle \lim _{F_{total}\rightarrow \infty }\lambda (F_{total})=\lambda _{min}>0, \quad \lambda (0)>\min \{\mu _f,\mu _{fw}\}, \quad \lambda _{min}<\min \{\mu _f,\mu _{fw}\}, \end{aligned}$$

hold. Then, the variables $(M,F,M_w,F_w)$ satisfying equations (2.2)–(2.5) remain non-negative if they are non-negative initially, and they remain bounded for all times.

#### Proof

First note that the solution variables remain non-negative for all time; this follows from results in Smith (1995). Adding equations (2.3) and (2.5), and noticing that $\beta \in [0,1]$, $q \in [0,1]$, we have

2.7 $$\begin{aligned} F_{total}^{\prime } \le&\displaystyle -\min \{\mu _f, \mu _{fw}\} F_{total} + \frac{\lambda (F_{total})}{M_{total} + F_{total}}M_{total}F_{total}\nonumber \\ \le&\displaystyle -\min \{\mu _f, \mu _{fw}\} F_{total} + \frac{\lambda (F_{total})}{M_{total} }M_{total}F_{total}\nonumber \\ =&\left( -\min \{\mu _f, \mu _{fw}\} + \lambda (F_{total})\right) F_{total}. \end{aligned}$$

Therefore,

$$\begin{aligned} \limsup _{t\rightarrow \infty } F_{total}(t) \le \bar{F} \end{aligned}$$

where $\bar{F}$ is such that $\lambda (\bar{F}) = \min \{\mu _f,\mu _{fw}\}$. Note $\bar{F}$ exists since we assumed $\lambda$ is monotone decreasing and satisfies (2.6).

Since $F_{total}$ remains bounded it follows that $M_{total}$ is bounded as well, because adding (2.2) and (2.4) we have

2.8 $$\begin{aligned} M_{total}^{\prime } \le&\displaystyle -\min \{\mu _m, \mu _{mw}\} M_{total} + \frac{\lambda (F_{total})}{M_{total} + F_{total}}M_{total}F_{total} \nonumber \\ \le&\displaystyle -\min \{\mu _m, \mu _{mw}\} M_{total} + \lambda (F_{total})F_{total} \nonumber \\ \le&-\min \{\mu _m, \mu _{mw}\} M_{total} + B, \end{aligned}$$

where *B* is an upper bound for $\lambda (F_{total}(t)) F_{total}(t)$, which exists since $F_{total}(t)$ is bounded and therefore so is $\lambda (F_{total}(t)) F_{total}(t)$. From the differential inequality (2.8), we can conclude that $M_{total}$ is bounded, too. $\square$

### Boundary equilibria and their stability

It is straightforward to see that model (2.2)–(2.5) has only one non-trivial *Wolbachia *free boundary equilibrium $E^*=(M^*, F^*,0,0)$, where $F^*$ satisfies

2.9 $$\begin{aligned} \lambda (F^*) = \mu _f +\mu _m, \end{aligned}$$

and

2.10 $$\begin{aligned} M^* = \frac{\mu _f F^*}{\mu _m}, \end{aligned}$$

under the assumptions that $\lambda (0) > \mu _f + \mu _m$, $\lambda$ is a decreasing non-negative function, and $\lambda (F_{total})\rightarrow \lambda _{min}$ (with $\lambda _{min}$ sufficiently small) as $F_{total} \rightarrow \infty$.

Note that there is no *Wolbachia *infected boundary equilibrium unless $\tau =1$, a case that we shall treat separately later.

#### Theorem 2.1

Suppose that $\lambda$ is a monotone decreasing non-negative function such that $\lambda (F_{total})\rightarrow \lambda _{min}$ as $F_{total} \rightarrow \infty$, with $\lambda _{min}$ sufficiently small, $\lambda (0)>\mu _f + \mu _m$ and

2.11 $$\begin{aligned} \frac{\mu _f (1-\beta )\tau }{\mu _{fw}} <1. \end{aligned}$$

Then, the *Wolbachia *free boundary equilibrium $E^*=(M^*, F^*,0,0)$ of model (2.2)–(2.5) is locally asymptotically stable.

#### Proof

Linearisation of system (2.2)–(2.5) at the equilibrium $E^*$ yields the following partially decoupled systems. The first system below is just the linearisation of equations (2.4)–(2.5) at the steady state, which we shall use to show that $(M_w(t),F_w(t)) \rightarrow (0,0)$ as $t \rightarrow \infty$. System (2.13) is just system (2.2)–(2.3) in the case $M_w = F_w =0$.

2.12 $$\begin{aligned}&\left\{ \begin{array}{lll} M_w^{\prime } &{} = &{}\displaystyle -\mu _{mw} M_w + \frac{\lambda (F^*)}{M^* + F^*} (1-\beta )\tau (1-\gamma )M^*F_w, \\ F_w^{\prime } &{} = &{}\displaystyle -\mu _{fw} F_w + \frac{\lambda (F^*)}{M^* + F^*} (1-\beta )\tau M^*F_w, \\ \end{array}\right. \end{aligned}$$

2.13 $$\begin{aligned}&\left\{ \begin{array}{lll} M^{\prime } &{} = &{}\displaystyle -\mu _{m} M + \frac{\lambda (F)}{M + F}MF, \\ F^{\prime } &{} = &{}\displaystyle -\mu _{f} F + \frac{\lambda (F)}{M + F}MF. \\ \end{array}\right. \end{aligned}$$

From the second equation of (2.12), it is clear that if

2.14 $$\begin{aligned} \frac{\lambda (F^*)}{M^* + F^*} (1-\beta )\tau M^* < \mu _{fw}, \end{aligned}$$

then $F_w(t) \rightarrow 0$ as $t \rightarrow \infty$. Then $M_w(t) \rightarrow 0$ as $t\rightarrow \infty$ follows from the first equation of (2.12). Since $M^*$ and $F^*$ are given by (2.9) and (2.10), inequality (2.14) is equivalent to assumption (2.11).

It remains to prove the local stability of $(M,F)=(M^*,F^*)$ as a solution of system (2.13). The Jacobian matrix of system (2.13) evaluated at $(M^*,F^*)$ is given by

$$\begin{aligned} J(M^*,F^*) = \frac{1}{\mu _f + \mu _m} \left( \begin{array}{l@{\quad }l} -\mu _f \mu _m &{} \mu _f^2 + \lambda ^{\prime }(F^*) F^* \mu _f \\ \mu _m^2 &{} -\mu _f \mu _m + \lambda ^{\prime }(F^*) F^* \mu _f \\ \end{array} \right) . \end{aligned}$$

The eigenvalues $\Lambda$ of $J(M^*,F^*)$ satisfy the characteristic equation

$$\begin{aligned} \Lambda ^2 + (2\mu _f \mu _m - \lambda ^{\prime }(F^*) F^* \mu _f)\Lambda - \lambda ^{\prime }(F^*) F^* (\mu _f+\mu _m)\mu _f\mu _m=0. \end{aligned}$$

Since $\lambda (\cdot )$ is a non-negative decreasing function, $\lambda ^{\prime }(F^*) <0$. We have

$$\begin{aligned} \Lambda _1 + \Lambda _2 = -(2\mu _f \mu _m - \lambda ^{\prime }(F^*) F^* \mu _f) <0, \end{aligned}$$

and

$$\begin{aligned} \Lambda _1 \Lambda _2 = - \lambda ^{\prime }(F^*) F^* (\mu _f+\mu _m)\mu _f\mu _m >0, \end{aligned}$$

which implies $\text{ Re }\,\Lambda _1 <0$ and $\text{ Re }\,\Lambda _2 <0$, so that $(M^*,F^*)$ is locally stable as a solution of (2.13). Therefore, the *Wolbachia *free equilibrium $E^*=(M^*,F^*,0,0)$ is locally asymptotically stable as a solution of the full system (2.2)–(2.5). $\square$

If $\tau =1$, i.e. we have complete maternal transmission of *Wolbachia*, then a boundary equilibrium of the form $(0,0,M_w^*,F_w^*)$ may exist. The components of such an equilibrium solution must satisfy

2.15 $$\begin{aligned} \begin{aligned}&\mu _{mw}M_w^*=(1-\gamma )\mu _{fw}F_w^*, \\&\mu _{fw}=\frac{\lambda (F_w^*)}{M_w^*+F_w^*}(1-\beta )M_w^*. \end{aligned} \end{aligned}$$

Moreover, $(1-\gamma )\mu _{fw}+\mu _{mw}=(1-\beta )(1-\gamma )\lambda (F_w^*)$ must hold. Next we study the linear stability of such equilibrium, showing that it is linearly stable under condition (2.16) below. Inequality (2.16) does not depend on $\gamma$, the male killing rate, but the steady state components $M_w^*$ and $F_w^*$ do depend on $\gamma$ in the manner expected (for example, $M_w^*=0$ when $\gamma =1$). Although Theorem 2.2 only apples if $\tau =1$, we will be interested later on in the case when $\tau$ is just slightly less than 1. Then, maternal transmission is imperfect and *Wolbachia *infected females produce small numbers of uninfected offspring. We anticipate that as $\tau$ decreases from 1 to a value just less than 1, the equilibrium $(0,0,M_w^*,F_w^*)$ shifts to another nearby position with small numbers of *Wolbachia *uninfected individuals and large numbers of infected ones; with no change of stability for $\tau$ close enough to 1. The existence and stability of such an equilibrium will be important later on when we introduce West Nile virus (WNv) disease dynamics because, at a WNv-free equilibrium with large numbers of *Wolbachia *infected mosquitoes, the basic reproduction number $R_0$ for WNv is likely to be less than 1. The implication is that *Wolbachia *infection in mosquitoes has the potential to control WNv infection. It does so by disrupting WNv virus replication causing WNv infected mosquitoes effectively to remain permanently (or for a very long time) in the latent stage of WNv.

#### Theorem 2.2

Assume that $\tau =1$, $\lambda$ is monotone decreasing with $\displaystyle \lim _{F_{total}\rightarrow \infty }\lambda (F_{total})=\lambda _{min}$, ($\lambda _{min}$ sufficiently small) and

$$\begin{aligned} \lambda (0)>\frac{(1-\gamma )\mu _{fw}+\mu _{mw}}{(1-\beta )(1-\gamma )} \end{aligned}$$

holds. Then, an equilibrium of the form $(M,F,M_w,F_w)=(0,0,M_w^*,F_w^*)$ exists, and it is locally stable as a solution of (2.2)–(2.5) if

2.16 $$\begin{aligned} (1-q)\mu _{fw}<(1-\beta )\mu _f. \end{aligned}$$

#### Proof

The proof is similar to that of Theorem 2.1. The linearisation around $(0,0,M_w^*,F_w^*)$ yields a system of linear equations for (*M*, *F*) that (when $\tau =1$) are decoupled from the rest of the system. Moreover, it may be shown that $(M,F)\rightarrow (0,0)$ as $t\rightarrow \infty$ if

$$\begin{aligned} \frac{(1-q)M_w^*\lambda (F_w^*)}{M_w^*+F_w^*}<\mu _f \end{aligned}$$

holds, which becomes inequality (2.16) when the equilibrium equations (2.15) are invoked. Then, the $F_w$ and $M_w$ equations are considered, in the case when $\tau =1$ and $F=M\equiv 0$. Tedious computations yield that the linearisation of that system around the steady state $(M_w^*,F_w^*)$ has the Jacobian matrix equal to $((1-\gamma )\mu _{fw}+\mu _{mw})^{-1}$ times

$$\begin{aligned} \left( \begin{array}{c@{\quad }c} -(1-\gamma )\mu _{fw}\mu _{mw} &{} (1-\beta )(1-\gamma )\left[ \left( \frac{1-\gamma }{1-\beta }\right) \mu _{fw}^2+\lambda '(F_w^*)F_w^*(1-\gamma )\mu _{fw}\right] \\ \mu _{mw}^2 &{} -\mu _{fw}\mu _{mw}+(1-\beta )\lambda '(F_w^*)F_w^*(1-\gamma )\mu _{fw} \end{array} \right) , \end{aligned}$$

and it may be further shown that its eigenvalues both have negative real parts. Thus we conclude that the steady state $(0,0,M_w^*,F_w^*)$ is locally asymptotically stable. $\square$

Next we prove that, under certain conditions, both infected and uninfected mosquitoes die out. Note, however, that (0, 0, 0, 0) is not technically an equilibrium of (2.2)–(2.5).

#### Theorem 2.3

Suppose that $\lambda$ is monotone decreasing and that $0<\lambda (F_{total})<\mu _f+\mu _m$ for all $F_{total}\ge 0$, and that

2.17 $$\begin{aligned} (\mu _f+\mu _m)(1-\beta )\tau <\mu _{fw}. \end{aligned}$$

Then $(M(t),F(t),M_w(t),F_w(t))\rightarrow (0,0,0,0)$ as $t\rightarrow \infty$ if all of the four variables are sufficiently small initially.

#### Proof

From (2.4) and (2.5),

2.18 $$\begin{aligned} M_w'(t)&\le -\mu _{mw}M_w(t)+(\mu _f+\mu _m)(1-\beta )\tau (1-\gamma )F_w(t), \end{aligned}$$

2.19 $$\begin{aligned} F_w'(t)&\le -\mu _{fw}F_w(t)+(\mu _f+\mu _m)(1-\beta )\tau F_w(t). \end{aligned}$$

From (2.19) and (2.17) we have $F_w(t)\rightarrow 0$ as $t\rightarrow \infty$. Then inequality (2.18) implies that $M_w(t)\rightarrow 0$ also. With $M_w=F_w=0$, we are reduced to system (2.13) and we now show that $(M(t), F(t))\rightarrow (0,0)$ as $t\rightarrow \infty$, though this result is local, i.e. for small introductions of *F* and *M*. Note that $(M,F)=(0,0)$ is not an equilibrium of (2.13), due to the singularity, but we can remove that singularity by introducing the new variable $\xi =F/M$. In terms of the variables $\xi$ and *M*, system (2.13) becomes

2.20 $$\begin{aligned} \begin{aligned} \xi '(t)&= -(\mu _f-\mu _m)\xi (t)+\left( \frac{1-\xi (t)}{1+\xi (t)}\right) \xi (t)\lambda (M(t)\xi (t)), \\ M'(t)&= -\mu _m M(t)+\frac{M(t)\xi (t)}{1+\xi (t)}\lambda (M(t)\xi (t)). \end{aligned} \end{aligned}$$

We now show that $(\xi ,M)=(\xi ^*,0)$ is a locally stable steady state of (2.20), where

2.21 $$\begin{aligned} \xi ^*=\frac{\lambda (0)+\mu _m-\mu _f}{\lambda (0)+\mu _f-\mu _m}, \end{aligned}$$

provided $\xi ^*>0$. The latter is not automatic. However, note that, from the first equation of (2.13), $M'(t)\le (\lambda (0)-\mu _m)M(t)$. Therefore, if $\lambda (0)<\mu _m$ then $M(t)\rightarrow 0$. The second of (2.13) then gives $F'(t)\le -\mu _f F(t)+\lambda (0)M(t)$ so that $F(t)\rightarrow 0$. By similar reasoning we arrive at the same conclusion if $\lambda (0)<\mu _f$. Therefore, we may assume henceforth that $\lambda (0)\ge \max (\mu _f,\mu _m)$ and, under these circumstances, $\xi ^*>0$. The linearisation of the second equation of (2.20) near the equilibrium $(\xi ,M)=(\xi ^*,0)$ reads

$$\begin{aligned} M'(t)=-\mu _m M(t)+\lambda (0)\frac{\xi ^*}{1+\xi ^*}M(t)=\frac{1}{2}(\lambda (0)-\mu _m-\mu _f)M(t), \end{aligned}$$

and therefore, since $\lambda (0)<\mu _m+\mu _f$, we have $M(t)\rightarrow 0$. In this limit the $\xi$ equation becomes

$$\begin{aligned} \xi '(t) = -(\mu _f-\mu _m)\xi (t)+\left( \frac{1-\xi (t)}{1+\xi (t)}\right) \xi (t)\lambda (0)=F(\xi (t)), \end{aligned}$$

and to show that $\xi ^*$ is locally stable as a solution of this equation, it suffices to show that $F'(\xi ^*)<0$. But, after some algebra, we have

$$\begin{aligned} F'(\xi ^*)=-\frac{(\lambda (0))^2-(\mu _m-\mu _f)^2}{2\lambda (0)}. \end{aligned}$$

To show that $F'(\xi ^*)<0$ holds, it suffices to show that $\lambda (0)>|\mu _m-\mu _f|$, i.e. that both $\lambda (0)>\mu _m-\mu _f$ and $\lambda (0)>\mu _f-\mu _m$ hold. But this follows from the fact that we are now restricting to the case when $\lambda (0)\ge \max (\mu _f,\mu _m)$. Therefore, the proof of the theorem is complete. $\square$

### Existence of strictly positive steady states

In this section we examine the possible existence of coexistence steady states $(M,F,M_w,F_w)$ of model (2.2)–(2.5), i.e. steady states in which each component is strictly positive. It turns out that in some parameter regimes multiple coexistence steady states may exist while, in others, there is just one or none at all. An understanding of these properties helps us to understand how one might exploit *Wolbachia *infection in mosquitoes to effectively control WNv. In this section we simplify by assuming that $\gamma =0$, i.e. that there is no male killing.

At the steady state, dividing (2.2) by (2.3), and (2.4) by (2.5), we obtain

2.22 $$\begin{aligned} M=F\frac{\mu _f}{\mu _m},\,\, M_w=F_w\frac{\mu _{fw}}{\mu _{mw}}. \end{aligned}$$

From (2.3) and (2.5) we then obtain

2.23 $$\begin{aligned} \mu _fF=&\frac{\lambda _*}{F\left( 1+\frac{\mu _f}{\mu _m}\right) +F_w\left( 1+\frac{\mu _{fw}}{\mu _{mw}}\right) } \nonumber \\&\times \left( \frac{\mu _f}{\mu _m}F^2+(1-\beta )(1-\tau )\left( \frac{\mu _f}{\mu _m}FF_w+\frac{\mu _{fw}}{\mu _{mw}}F_w^2\right) \right. \nonumber \\&\quad \left. +(1-q)\frac{\mu _{fw}}{\mu _{mw}}FF_w\right) , \end{aligned}$$

2.24 $$\begin{aligned} \mu _{fw}F_w=&\frac{\lambda _*}{F\left( 1+\frac{\mu _f}{\mu _m}\right) +F_w\left( 1+\frac{\mu _{fw}}{\mu _{mw}}\right) }\left( (1-\beta )\tau \left( \frac{\mu _f}{\mu _m}FF_w+\frac{\mu _{fw}}{\mu _{mw}}F_w^2\right) \right) , \end{aligned}$$

respectively, where $\lambda _*=\lambda (F+F_w)$. From (2.24) we have

2.25 $$\begin{aligned}&F_w\left( \mu _{fw}+\frac{\mu ^2_{fw}}{\mu _{mw}}\right) +F\left( \mu _{fw}+\frac{\mu _{fw}\mu _f}{\mu _m}\right) \nonumber \\&\quad =\lambda _* \left( F(1-\beta )\tau \frac{\mu _f}{\mu _m}+F_w(1-\beta )\tau \frac{\mu _{fw}}{\mu _{mw}}\right) . \end{aligned}$$

From (2.25) we obtain

2.26 $$\begin{aligned} F_w\kappa _1(\lambda _*)=F\kappa _2(\lambda _*), \end{aligned}$$

where

2.27 $$\begin{aligned} \kappa _1(\lambda _*)= & {} \mu _{fw}+\frac{\mu _{fw}^2}{\mu _{mw}}-\lambda _*(1-\beta )\tau \frac{\mu _{fw}}{\mu _{mw}},\nonumber \\ \kappa _2(\lambda _*)= & {} -\mu _{fw}-\mu _{fw}\frac{\mu _f}{\mu _m}+\lambda _*(1-\beta )\tau \frac{\mu _{f}}{\mu _{m}}. \end{aligned}$$

Note that if at the strictly positive steady state, we have $\kappa _1(\lambda _*)=0$, then this necessarily implies that $\kappa _2(\lambda _*)=0$. This is only possible if

2.28 $$\begin{aligned} \frac{\mu _{fw}}{\mu _f}=\frac{\mu _{mw}}{\mu _m} \end{aligned}$$

holds. This case is excluded from Theorem 2.4 but is treated in the next subsection.

If (2.28) does not hold then, using (2.27), from (2.23) we obtain

2.29 $$\begin{aligned} 0&= \kappa ^2_1(\lambda _*)\left( \mu _f+\frac{\mu ^2_f}{\mu _m}-\lambda _*\frac{\mu _f}{\mu _m}\right) -\kappa ^2_2(\lambda _*)\lambda _*(1-\beta )(1-\tau )\frac{\mu _{fw}}{\mu _{mw}} \nonumber \\&\quad +\kappa _1(\lambda _*)\kappa _2(\lambda _*)\left( \mu _f+\frac{\mu _f\mu _{fw}}{\mu _{mw}}-\lambda _*(1-\beta )(1-\tau )\frac{\mu _f}{\mu _m}-\lambda _*(1-q)\frac{\mu _{fw}}{\mu _{mw}}\right) . \end{aligned}$$

The right hand side of (2.29) is, in general, a cubic polynomial in $\lambda _*$. If there exists a positive root $\lambda ^1_*$, then since $\lambda$ is a strictly monotone function, a corresponding unique $F^1+F^1_w$ value may be found. From (2.23) we may then determine a unique solution $(F^1,F^1_w)$. Further analytic progress is possible in certain particular cases of interest, which we now investigate. The first concerns the case when $\tau =1$, or when $\tau$ is very close to 1, meaning that maternal transmission of *Wolbachia *is complete or nearly complete. This is in fact the biologically relevant case for a number of CI inducing *Wolbachia *strains in mosquito species treated in the literature, see e.g. Engelstädter et al. (2004). This leads us to expect the existence of a steady state of (2.2)–(2.5) with large numbers of *Wolbachia *infected mosquitoes and few, or no, uninfected ones. The stability of such a steady state still depends on the other parameter values, and it will be stable if there is a high probability of mating between infected males and uninfected females resulting in no offspring (the effect of CI), i.e. *q* is close to 1; see also inequality (2.16) for the case $\tau =1$. The existence of a stable steady state of (2.2)–(2.5) with the above mentioned properties is important because the low number of *Wolbachia *uninfected females implies that the quantity $F_s^*$, featuring in the first term of the parameter $R_0$ defined later in (3.47), is small. The likelihood of $R_0$ being less than 1 (the condition for WNv-eradication) depends mostly on that first term involving $F_s^*$, since the second term in (3.47) involves a small parameter $\varepsilon$ and is automatically small. For these reasons, we are interested in stable steady states of model (2.2)–(2.5) of the form $(M^*,F^*,M_w^*,F_w^*)$, with $M^*$ and $F^*$ small compared to $M_w^*$ and $F_w^*$ (and, ideally, $M^*=F^*=0$). Therefore, referring to Theorems 2.1 and 2.2, and restricting for now to the case $\tau =1$, we ideally would like the boundary equilibrium $(M^*,F^*,0,0)$ of Theorem 2.1 to be unstable, and the boundary equilibrium $(0,0,M_w^*,F_w^*)$ of Theorem 2.2 to be linearly stable. The conditions for this, when $\tau =1$, are that the inequalities $\mu _f(1-\beta )>\mu _{fw}$ and $(1-q)\mu _{fw}<(1-\beta )\mu _f$ should hold simultaneously, but note that the second of these follows from the first. For $\tau =1$, guided by elementary competition theory, instability of one boundary equilibrium and stability of the other suggests that there will be no coexistence equilibrium, and this is what we prove in Theorem 2.4 below. If $\tau$ is decreased from 1 to a value slightly below 1, there is no longer a boundary equilibrium with only *Wolbachia *infected mosquitoes present. What happens is that the equilibrium $(0,0,M_w^*,F_w^*)$, which exists when $\tau =1$, moves to another nearby point in $\mathbb {R}^4_+$, so that *Wolbachia *infected mosquitoes now coexist with uninfected ones, the former being dominant. Very importantly, this will be the only coexistence steady state if $\tau$ is sufficiently close to 1, and it is a desirable steady state for WNv eradication because particular *Wolbachia *strains can significantly reduce WNv virus replication in mosquitoes, see Hussain (2013).

#### Theorem 2.4

Suppose that $\gamma =0$, $\tau =1$, $\mu _f(1-\beta )>\mu _{fw}$, $\mu _m\mu _{fw}\ne \mu _f\mu _{mw}$, and that $\lambda$ is a strictly positive decreasing function with $\lambda (\infty )=\lambda _{min}$ (and $\lambda _{min}$ sufficiently small). Then system (2.2)–(2.5) has no coexistence equilibrium with $M^*,F^*,M_w^*,F_w^*>0$.

If the foregoing hypotheses hold, except that $\tau$ is slightly less than 1, then system (2.2)–(2.5) has precisely one coexistence equilibrium in which *Wolbachia *uninfected mosquitoes exist in very small numbers relative to *Wolbachia *infected ones.

#### Proof

Since we assume $\tau =1$, the form of (2.29) simplifies and in fact we may cancel $\kappa _1(\lambda _*)$ since we seek equilibria in which $M^*,F^*,M_w^*,F_w^*>0$. After some further algebra, we find that there is just one value for $\lambda _*$, given by

2.30 $$\begin{aligned} \begin{aligned} \lambda _*\,q(1-\beta )\frac{\mu _{fw}\mu _f}{\mu _{mw}\mu _m}&= (1-\beta )\left( \frac{\mu _f\mu _{fw}}{\mu _{mw}}-\frac{\mu _f^2}{\mu _m}\right) +q\frac{\mu _f\mu _{fw}^2}{\mu _m\mu _{mw}} \\&\quad \,\, +\frac{\mu _f\mu _{fw}}{\mu _m}-(1-q)\frac{\mu _{fw}^2}{\mu _{mw}}. \end{aligned} \end{aligned}$$

If $\lambda _*\le 0$ then the equation $\lambda _*=\lambda (F+F_w)$ cannot be solved for $F+F_w$, so we may restrict to the case that $\lambda _*>0$. Recalling that $\kappa _1$ and $\kappa _2$ are defined by (2.27), we find, with $\lambda _*$ given by (2.30) and with $\tau =1$, that

2.31 $$\begin{aligned} \kappa _1(\lambda _*)=\left( 1-\frac{\mu _m\mu _{fw}}{\mu _f\mu _{mw}}\right) \left[ \mu _{fw}+\frac{1}{q}\big ((1-\beta )\mu _f-\mu _{fw}\big )\right] , \end{aligned}$$

and

2.32 $$\begin{aligned} \kappa _2(\lambda _*)=\frac{1}{q}\big ((1-\beta )\mu _f-\mu _{fw}\big )\left( 1-\frac{\mu _f\mu _{mw}}{\mu _m\mu _{fw}}\right) . \end{aligned}$$

Since $\mu _f(1-\beta )>\mu _{fw}$ it follows that the sign of the product $\kappa _1(\lambda _*)\kappa _2(\lambda _*)$ is the same as the sign of

$$\begin{aligned} \left( 1-\frac{\mu _m\mu _{fw}}{\mu _f\mu _{mw}}\right) \left( 1-\frac{\mu _f\mu _{mw}}{\mu _m\mu _{fw}}\right) \end{aligned}$$

and, since we assume $\mu _m\mu _{fw}\ne \mu _f\mu _{mw}$, it follows that $\kappa _1(\lambda _*)\kappa _2(\lambda _*)<0$. This makes it impossible to find $F>0$ and $F_w>0$ satisfying (2.26), and so there is no coexistence equilibrium.

Next we prove the second assertion of the theorem. Let $\tau =1-\varepsilon$. Since we expect the equilibrium $(0,0,M_w^*,F_w^*)$, which exists when $\tau =1$, to move to another nearby point when $\tau =1-\epsilon$, for $\epsilon$ sufficiently small, we seek an equilibrium of (2.2)–(2.5) of the form

$$\begin{aligned} M(\varepsilon )=&\varepsilon M^{(1)}+\varepsilon ^2 M^{(2)}+\cdots , \\ F(\varepsilon )=&\varepsilon F^{(1)}+\varepsilon ^2 F^{(2)}+\cdots , \\ M_w(\varepsilon )=&M_w^*+\varepsilon M_w^{(1)}+\varepsilon ^2 M_w^{(2)}+\cdots , \\ F_w(\varepsilon )=&F_w^*+\varepsilon F_w^{(1)}+\varepsilon ^2 F_w^{(2)}+\cdots . \end{aligned}$$

Coefficients of $\varepsilon$ yield

2.33 $$\begin{aligned} \mu _m M^{(1)}=\frac{\lambda (F_w^*)}{M_w^*+F_w^*}\left[ (1-\beta )M_w^*F_w^*+(1-q)M_w^*F^{(1)}\right] , \end{aligned}$$

and

2.34 $$\begin{aligned} \mu _f F^{(1)}=\frac{\lambda (F_w^*)}{M_w^*+F_w^*}\left[ (1-\beta )M_w^*F_w^*+(1-q)M_w^*F^{(1)}\right] . \end{aligned}$$

Using

$$\begin{aligned} \mu _{fw}=\frac{\lambda (F_w^*)}{M_w^*+F_w^*}(1-\beta )M_w^*, \end{aligned}$$

(2.34) reads

$$\begin{aligned} \mu _fF^{(1)}=\mu _{fw}F_w^*+\frac{\mu _{fw}(1-q)}{1-\beta }F^{(1)}, \end{aligned}$$

so that

2.35 $$\begin{aligned} F^{(1)}=\frac{(1-\beta )\mu _{fw}F_w^*}{(1-\beta )\mu _f-(1-q)\mu _{fw}}. \end{aligned}$$

Note that $F^{(1)}>0$ because of the assumption $\mu _f(1-\beta )>\mu _{fw}$, hence from (2.33) we conclude that $M^{(1)}>0$ holds, and

2.36 $$\begin{aligned} M^{(1)}=\frac{\mu _{fw}\mu _f(1-\beta )F_w^*}{\mu _m\left[ (1-\beta )\mu _f-(1-q)\mu _{fw}\right] }. \end{aligned}$$

In conclusion, if $\tau$ is reduced from 1 to the value $1-\varepsilon$ then the equilibrium $(0,0,M_w^*,F_w^*)$ moves to $(\varepsilon M^{(1)},\varepsilon F^{(1)},M_w^*+\varepsilon M_w^{(1)},F_w^*+\varepsilon F_w^{(1)})$, with $M^{(1)}$ and $F^{(1)}$ given by (2.36) and (2.35), respectively. $\square$

Note that from the proof of Theorem 2.4 we can see that at the equilibrium the male/female ratio for *Wolbachia *uninfected mosquitoes is given approximately by $\frac{\mu _f}{\mu _m}$.

Theorem 2.4 excludes the case when $\mu _m\mu _{fw}=\mu _f\mu _{mw}$, which we treat now. In particular, we assume that $\gamma =0$, and $\frac{\mu _{f}}{\mu _m}=\mu =\frac{\mu _{fw}}{\mu _{mw}}$. In this situation, we have $M=\mu F$, and $M_w=\mu F_w$. From equations (2.3) and (2.5) we obtain

2.37 $$\begin{aligned} \mu _fF=&\frac{\mu \lambda _*}{(1+\mu )(F+F_w)}\left( F^2+(1-\beta )(1-\tau )\left( FF_w+F^2_w\right) +(1-q)FF_w\right) , \end{aligned}$$

2.38 $$\begin{aligned} \mu _{fw}F_w=&\frac{\mu \lambda _*}{(1+\mu )(F+F_w)}\left( (1-\beta )\tau \left( FF_w+F^2_w\right) \right) . \end{aligned}$$

From equation (2.38) we find that

2.39 $$\begin{aligned} \lambda _*=\lambda (F+F_w)=\frac{(1+\mu )\mu _{fw}}{\mu (1-\beta )\tau }, \end{aligned}$$

hence for the existence of a coexistence steady state it is necessary that $\lambda (0)>\frac{(1+\mu )\mu _{fw}}{\mu (1-\beta )\tau }$, in which case there exists a unique $c=F+F_w$, such that $\lambda (c)=\frac{(1+\mu )\mu _{fw}}{\mu (1-\beta )\tau }$ holds. Then, from equation (2.37) we obtain

2.40 $$\begin{aligned} \frac{\mu _f}{\mu _{fw}}c(1-\beta )\tau F=F^2+(1-q)FF_w+c(1-\beta )(1-\tau )F_w, \end{aligned}$$

from which, using $F_w=c-F$, we obtain

2.41 $$\begin{aligned} 0=F^2q+Fc\left( (1-q)-(1-\beta )(1-\tau )-(1-\beta )\tau \frac{\mu _f}{\mu _{fw}}\right) +c^2(1-\beta )(1-\tau ). \end{aligned}$$

For the existence of the positive steady state we need to guarantee that the quadratic equation above has (at least one) positive (real) solution, and that the solution is less than *c*.

From equation (2.41) it is clear that in case of complete maternal transmission, i.e. for $\tau =1$ there cannot be more than one coexistence steady state. In this case, from equation (2.41) we obtain

2.42 $$\begin{aligned} F=\frac{c\left( (1-\beta )\frac{\mu _f}{\mu _{fw}}-(1-q)\right) }{q}. \end{aligned}$$

Therefore, if

2.43 $$\begin{aligned} 1-q<(1-\beta )\frac{\mu _f}{\mu _{fw}}<1 \end{aligned}$$

holds, then we have $0<F<c$, and if $\lambda (0)>\frac{(1+\mu )\mu _{fw}}{\mu (1-\beta )\tau }$ also holds, then a unique strictly positive steady state exists.

Circumstances under which (2.43) is likely to hold include that *q* is sufficiently close to 1 and, at the same time, $\beta$ is sufficiently close to 1 or $\frac{\mu _f}{\mu _{fw}}$ is less than 1. The biological interpretation is clear: for the existence of a coexistence steady state, the fertility cost (or mortality increase) due to *Wolbachia *infection should be sufficiently large.

It is clear from (2.41) that we can never have more than two coexistence steady states. On the other hand it is interesting to show that for some realistic parameter values it is possible to have two coexistence steady states.

To this end we consider the case $q=1$, $\frac{\mu _f}{\mu _{fw}}=1$, and we assume that $\tau \ne 1$, $\beta \ne 1$. In this case, from (2.41), we have

2.44 $$\begin{aligned} F_{1/2}=c\frac{1-\beta }{2}\left( 1\pm \sqrt{1-\frac{4(1-\tau )}{1-\beta }}\right) . \end{aligned}$$

That is, for $0<F_{1/2}<c$ to hold, we need to assume that

2.45 $$\begin{aligned} 4(1-\tau )<1-\beta ,\quad 1+\sqrt{1-\frac{4(1-\tau )}{1-\beta }}<\frac{2}{1-\beta } \end{aligned}$$

hold simultaneously. It is easy to verify that this can be achieved for any *c*, for example with $\tau =0.99$, $\beta =0.5$. Note that the condition $\frac{\mu _f}{\mu _{fw}}=1$ can be relaxed, too. Also, by continuity arguments, it follows that for parameter values close enough we still have two coexistence steady states. We summarise our findings in the following proposition.

#### Proposition 2.2

In the case when $\gamma =0$, $q=1$ and $\frac{\mu _f}{\mu _m}=\frac{\mu _{fw}}{\mu _{mw}}$, there exists a set of values for the remaining parameters such that system (2.2)–(2.5) admits two coexistence steady states.

In summary, we have shown that our *Wolbachia* model (2.2)–(2.5) may exhibit all of the three qualitatively different possible scenarios, i.e. when there are 0, 1 or 2 coexistence steady states.

## Model incorporating West Nile virus (WNv)

There has been considerable recent interest in West Nile virus (WNv), with the great majority of mathematical papers on the topic having appeared in the last 15 years. Numerous types of models have appeared, some including spatial effects and others giving consideration to issues such as age-structure in hosts, optimal control or backward bifurcation. See, for example, Blayneh et al. (2010), Bowman et al. (2005), Gourley et al. (2006/07), Lewis et al. (2006) and Wonham and Lewis (2008).

We introduce the following model as an extension of model (2.2)–(2.5) to include WNv dynamics. Our model for WNv dynamics has similarities to that in Bergsman et al. (2016) with one resident bird population. Hence, as in Bergsman et al. (2016) and in some references therein, we compartmentalise the vector and bird population into SEI and SEIR classes, respectively. Taking into account our earlier model the complete WNv-*Wolbachia* mosquito-bird population model takes the following form.

3.46 $$\begin{aligned} F_s^{\prime }&= \displaystyle \frac{\lambda (F_{total})}{M+ M_w + F + F_w} (MF + (1-\beta )(1-\tau )(MF_w + M_wF_w) \nonumber \\&\quad + (1-q)M_wF ) - \mu _f F_s - \alpha _f p_{bf} F_s \frac{B_i}{B_{total}}, \nonumber \\ F_e^{\prime }&= \displaystyle \alpha _f p_{bf} F_s \frac{B_i}{B_{total}} -\mu _f F_e - \nu _f F_e, \nonumber \\ F_i^{\prime }&= \displaystyle \nu _f F_e - \mu _f F_i,\nonumber \\ F_{ws}^{\prime }&= \displaystyle \frac{\lambda (F_{total})}{M+ M_w + F + F_w} (1-\beta )\tau (MF_w + M_wF_w) - \mu _{fw} F_{ws} \nonumber \\&\quad - \alpha _{fw} p_{bf} F_{ws} \frac{B_i}{B_{total}},\nonumber \\ F_{we}^{\prime }&= \displaystyle \alpha _{fw} p_{bf} F_{ws} \frac{B_i}{B_{total}} -\mu _{fw} F_{we} - \varepsilon \nu _f F_{we}, \nonumber \\ F_{wi}^{\prime }&= \displaystyle \varepsilon \nu _{f} F_{we} - \mu _{fw} F_{wi},\nonumber \\ B_s^{\prime }&= \displaystyle \Pi (B_{total} ) - \mu _b B_s - \left( \alpha _f p_{fb}F_i \frac{B_s}{B_{total}} + \alpha _{fw} p_{fb} F_{wi} \frac{B_s}{B_{total}} \right) ,\nonumber \\ B_e^{\prime }&= \displaystyle \alpha _f p_{fb}F_i \frac{B_s}{B_{total}} + \alpha _{fw} p_{fb} F_{wi} \frac{B_s}{B_{total}} - \mu _b B_e - \nu _b B_e, \nonumber \\ B_i^{\prime }&= \displaystyle \nu _b B_e - \mu _b B_i - \mu _{bi}B_i - \nu _i B_i,\nonumber \\ B_r^{\prime }&= \displaystyle \nu _iB_i - \mu _b B_r,\nonumber \\ M^{\prime }&= \displaystyle \frac{\lambda (F_{total})}{M+ M_w + F + F_w} (MF \nonumber \\&\quad + (1-\beta )(1-\tau )(MF_w + M_wF_w) + (1-q)M_wF ) - \mu _m M,\nonumber \\ M_w^{\prime }&= \displaystyle \frac{\lambda (F_{total})}{M+ M_w + F + F_w} (1-\beta )\tau (1-\gamma )(MF_w + M_wF_w) - \mu _{mw} M_w, \end{aligned}$$

where

$$\begin{aligned} B_{total}&= B_s +B_e +B_i +B_r, \\ F_{total}&= F+ F_w,\quad F_w = F_{ws} + F_{we} + F_{wi},\quad F = F_s + F_e + F_i. \end{aligned}$$

Some of the parameters of system (3.46) are defined after (2.2)–(2.5); the rest are defined as follows:

- $\alpha _f$: biting rate of female *Wolbachia *uninfected mosquitoes;
- $\alpha _{fw}$: biting rate of female *Wolbachia *infected mosquitoes;
- $p_{bf}$: transmission probability of WNv from infectious birds to WNv-susceptible female mosquitoes;
- $p_{fb}$: transmission probability of WNv from WNv-infectious female mosquitoes to susceptible birds;
- $\nu _f$: per-capita transition rate of WNv-exposed female *Wolbachia *uninfected mosquitoes to the infectious stage of WNv;
- $\varepsilon \in [0,1]$: small parameter modelling increased time that *Wolbachia *infected mosquitoes spend in the latent stage of WNv, due to the tendency of *Wolbachia *infection to hamper the replication of WNv in mosquitoes;
- $\nu _b$: per-capita transition rate of WNv-exposed birds to the infectious stage of WNv;
- $\nu _i$: per-capita rate at which infectious birds recover;
- $\mu _b$: per-capita natural death rate for birds;
- $\mu _{bi}$: per-capita WNv-induced death rate for infectious birds.

In this section, it must be emphasized that we are considering two different kinds of infection. Mosquitoes may be infected by either *Wolbachia *or WNv, or both. WNv infection is assumed possible only for female mosquitoes (since it is females that bite) and is modelled using an SEI (susceptible-exposed-infectious) approach with subscripts *s*, *e* and *i*. The variables $F_s$, $F_e$ and $F_i$ denote the numbers of *Wolbachia *uninfected mosquitoes that have susceptible, exposed and infectious status with respect to WNv. A subscript *w* indicates *Wolbachia *infection, so that $F_{ws}$, $F_{we}$ and $F_{wi}$ denote the numbers of *Wolbachia *infected mosquitoes that have susceptible, exposed and infectious status with respect to WNv. The variables *M* and $M_w$ are the numbers of *Wolbachia *uninfected and *Wolbachia *infected male mosquitoes, none of which have WNv. Birds are only susceptible to WNv and their numbers are given by the variables $B_s$, $B_e$, $B_i$ and $B_r$ denoting susceptible, exposed, infectious and recovered birds. Many of the terms of system (3.46) are also present in (2.2)–(2.5) without change, here we just discuss the extra terms that model the addition of WNv dynamics. WNv-susceptible mosquitoes, whether *Wolbachia *infected or not, acquire WNv infection by biting infectious birds; this is modelled via the last term in the first and fourth equations of (3.46) using the idea of mass action normalised by total host density, the biting rates (the $\alpha$ parameters defined above) having been separated out, rather than being absorbed into the transmission coefficients $p_{bf}$ and $p_{fb}$ as is often customary. Having acquired WNv infection from a bird, a mosquito enters the latent phase of WNv and is classed as an exposed mosquito. Exposed mosquitoes become WNv-infectious at rates $\nu _f F_e$ and $\varepsilon \nu _f F_{we}$ for *Wolbachia *uninfected and *Wolbachia *infected mosquitoes, respectively. In the latter, the presence of $\varepsilon \in [0,1]$ models the tendency of *Wolbachia *infected mosquitoes that have contracted WNv infection to spend a greater amount of time in the latent stage of WNv, since *Wolbachia *infection tends to block WNv replication making it less likely that such a mosquito would ever become WNv-infectious. Of course, we are at liberty to take $\varepsilon$ very small indeed, with the implication that the *Wolbachia *infected mosquito spends so long in the latent stage of WNv that it probably dies in that stage. This is our approach to modelling the blocking of WNv replication by *Wolbachia*.

Birds acquire WNv from bites by WNv-infectious mosquitoes, which may or may not have *Wolbachia *infection as well. Thus there are two infection rates for birds, these can be found in the right hand side of the seventh equation of (3.46), and also in the eighth equation since birds initially enter the exposed stage of WNv. This has a mean duration of $1/\nu _b$ for birds, after which they become WNv-infectious. Birds may recover from WNv, at a per-capita rate $\nu _i$. Note that, for birds, death due to WNv is modelled using a separate parameter $\mu _{bi}$ to distinguish from natural death, accounted for by $\mu _b$. The function $\Pi (B_{total})$ is the birth rate function for birds.

The approach we use here to model the latency stage of WNv (in either birds or mosquitoes) is not the only possible approach. Our approach permits individuals to spend different amounts of time in the latency stage, and we may only speak of the mean time spent in that stage. There are other approaches in which all individuals of a particular status (for example, all *Wolbachia *uninfected mosquitoes) spend the same amount of time in the latent stage of WNv. The time could be different for *Wolbachia *infected mosquitoes. These approaches result in models with time delays.

### Local stability of the WNv-free equilibria

Equilibria of system (3.46) may exist in which WNv is absent. Such WNv-free equilibria include the equilibrium $(M^*,F^*,0,0)$ considered in Theorem 2.1, in which both WNv and *Wolbachia *are absent, and equilibria in which WNv is absent but *Wolbachia *are present. We show that multiple WNv-free equilibria may coexist that have both *Wolbachia *uninfected and *Wolbachia *infected mosquitoes, we present a necessary and sufficient condition for any particular WNv-free equilibrium to be locally stable, and we show that the most likely scenario for eradication of WNv is to have large numbers of *Wolbachia *infected mosquitoes, with solutions of system (3.46) evolving to a WNv-free equilibrium that has large numbers of *Wolbachia *infected mosquitoes and relatively few uninfected ones. Theorem 3.1 applies to any WNv-free equilibrium, of which there may be several. Of course, we may have $R_0<1$ at one WNv-free equilibrium and $R_0>1$ at another. It depends on the values of $F_s^*$, $F_{ws}^*$ and $B_s^*$ for the particular WNv-free equilibrium under consideration. For clarity of exposition, we include as a hypothesis that the equilibrium be stable to the subset of perturbations in which WNv is absent (i.e. stable as a solution of the subsystem (2.2)–(2.5)), rather than including explicit conditions for stability of an equilibrium as a solution of that subsystem. The latter stability problem is a tedious one in its own right and is under consideration elsewhere in this paper. Theorem 3.1, in the form presented below, highlights clearly the particular role played by $R_0$.

#### Theorem 3.1

Let $F_s^*$, $F_{ws}^*$ and $B_s^*$ be the equilibrium values for the female susceptible (to WNv) *Wolbachia *uninfected and *Wolbachia *infected mosquitoes and susceptible birds, in any WNv-free equilibrium. Let

3.47 $$\begin{aligned} R_0=\frac{\nu _b\nu _fp_{fb}p_{bf}}{(\mu _b+\mu _{bi}+\nu _i)(\mu _b+\nu _b)}\left( \frac{\alpha _f^2(F_s^*/B_s^*)}{\mu _f(\mu _f+\nu _f)}+ \frac{\varepsilon \alpha _{fw}^2(F_{ws}^*/B_s^*)}{\mu _{fw}(\mu _{fw}+\epsilon \nu _f)}\right) . \end{aligned}$$

Then, if $R_0<1$, the WNv-free equilibrium under consideration is locally stable as a solution of the full system (3.46), if it is stable to perturbations in which the exposed and infectious variables remain zero.

#### Proof

At any WNv-free equilibrium, the linearisation of system (3.46) decouples to some extent making it sufficient to show that, when $R_0<1$, each component of the solution of the following system:

3.48 $$\begin{aligned} F_e'= & {} \frac{\alpha _fp_{bf}F_s^*}{B_s^*}B_i-(\mu _f+\nu _f)F_e, \nonumber \\ F_i'= & {} \nu _f F_e - \mu _f F_i, \nonumber \\ F_{we}'= & {} \frac{\alpha _{fw}p_{bf}F_{ws}^*}{B_s^*} B_i-(\mu _{fw}+\varepsilon \nu _f)F_{we}, \nonumber \\ F_{wi}'= & {} \varepsilon \nu _f F_{we}-\mu _{fw}F_{wi}, \nonumber \\ B_e'= & {} \alpha _f p_{fb}F_i+\alpha _{fw}p_{fb}F_{wi}-(\mu _b+\nu _b)B_e, \nonumber \\ B_i'= & {} \nu _b B_e - (\mu _b+\mu _{bi}+\nu _i)B_i, \end{aligned}$$

tends to zero. It is taken as a hypothesis that the susceptible variables then approach their respective steady state values. Note that system (3.48) has a structure that allows the application of Theorem 5.5.1 in Smith (1995), making it possible to restrict attention to the real roots of the characteristic equation associated with (3.48). That characteristic equation, corresponding to trial solutions with temporal dependence $\exp (\lambda t)$, is most easily analysed when written in the form

3.49 $$\begin{aligned}&(\lambda +\mu _b+\mu _{bi}+\nu _i)(\lambda +\mu _b+\nu _b)B_s^* \nonumber \\&\quad = \nu _b\nu _fp_{fb}p_{bf}\left[ \frac{\alpha _f^2F_s^*}{(\lambda +\mu _f)(\lambda +\mu _f+\nu _f)}+\frac{\varepsilon \alpha _{fw}^2F_{ws}^*}{(\lambda +\mu _{fw})(\lambda +\mu _{fw}+\epsilon \nu _f)}\right] . \nonumber \\ \end{aligned}$$

As functions of the real variable $\lambda$, the right hand side of (3.49) is decreasing, at least for $\lambda \ge 0$, while the left hand side is a quadratic with two real negative roots. A simple graphical argument shows that if the left hand side exceeds the right hand side when $\lambda =0$ (i.e., if $R_0<1$, with $R_0$ defined by (3.47)), then any real roots of the characteristic equation are negative which, since only the real roots need to be considered, implies that each component of the solution of (3.48) approaches zero as $t\rightarrow \infty$. The proof of the theorem is now complete. $\square$

## Numerical simulations

**Fig. 1:**
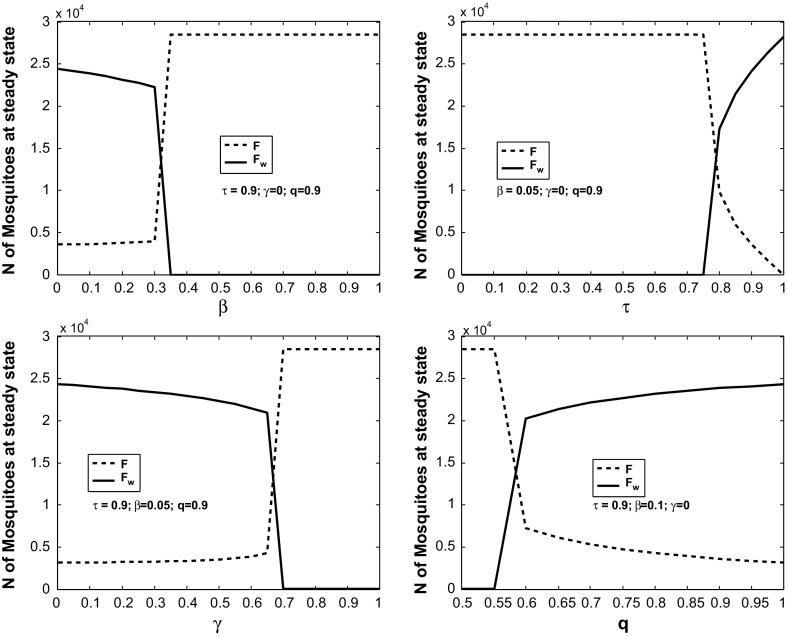
The dependence of the values of *F* and $F_w$ at the steady state of model (2.2)–(2.5) on the parameters $\beta , \tau , \gamma$ and *q*. Here, all the per-capita mortality rates of mosquitoes were taken as 0.05, and we set $r=30$, $k=5000$

**Fig. 2:**
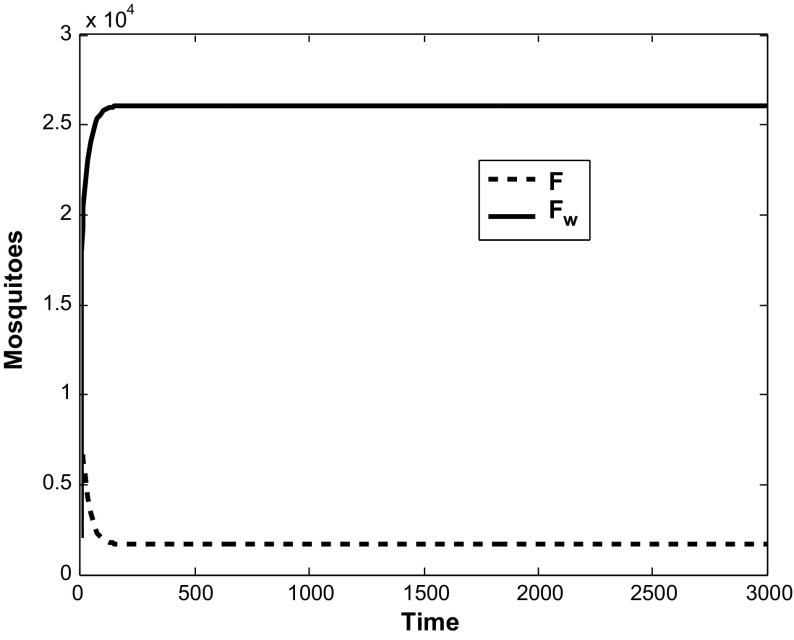
Simulation of model (2.2)–(2.5) showing *F* and $F_w$ against time. Here, all the per-capita mortality rates of mosquitoes are taken as 0.05, and we set $r = 30$, $k = 5000$, $\beta = 0.1$, $q = 0.9$, $\gamma = 0$ and $\tau = 0.95$

**Fig. 3:**
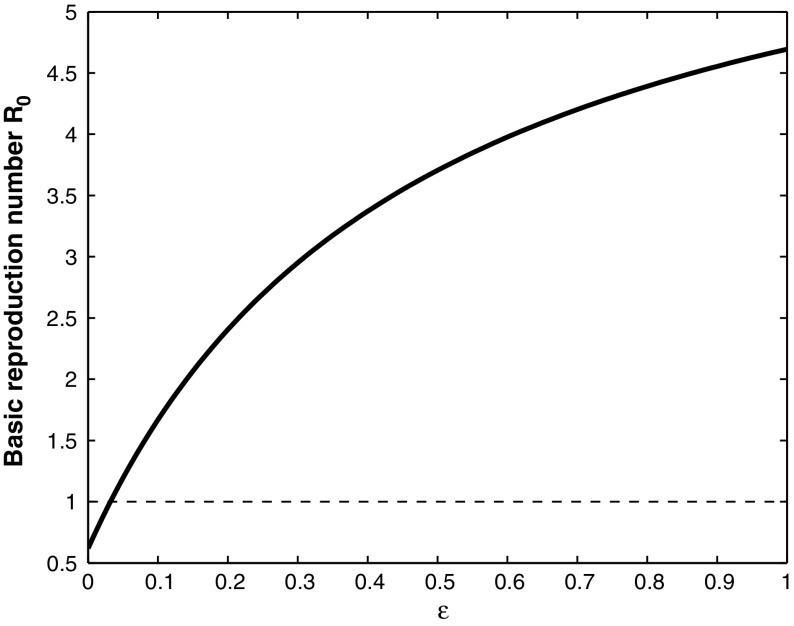
The basic reproduction number $R_0$, defined in (3.47), plotted against $\varepsilon$. The parameter values are $\beta = 0.1$, $q = 0.9$, $\gamma = 0$, $\tau = 0.9$, with the other parameter values given in Table 1. For these parameter values, $F_s^* = 3630$, $F_{ws}^* = 23837$, $B_s^* = 300$, and almost all of the mosquitoes are infected with *Wolbachia*. In this case, if $\varepsilon <0.03$, the basic reproduction number $R_0<1$, and the WNv will die out

**Fig. 4:**
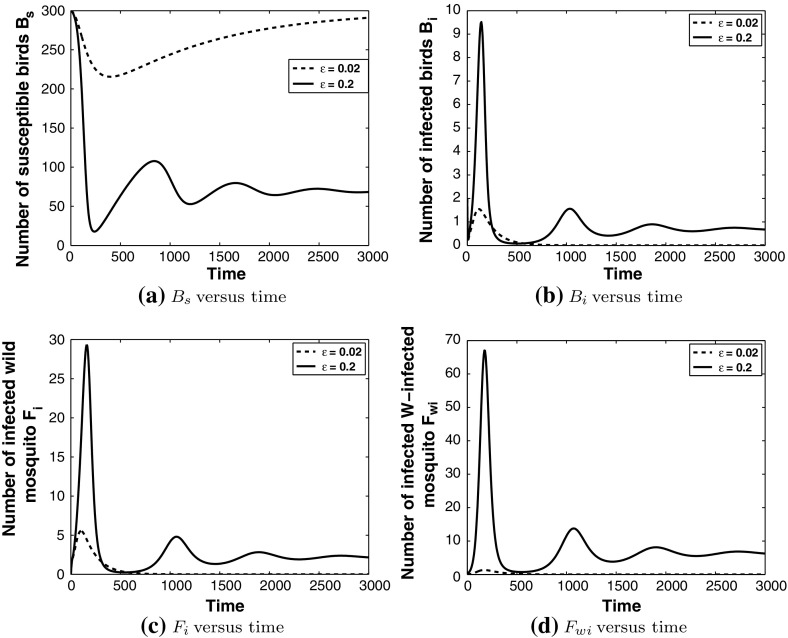
Simulation of model (3.46) with the parameter values as given in the caption of Fig.  3 for the cases $\varepsilon =0.2$ and $\varepsilon =0.02$. In the case $\varepsilon =0.02$, WNv dies out

**Fig. 5:**
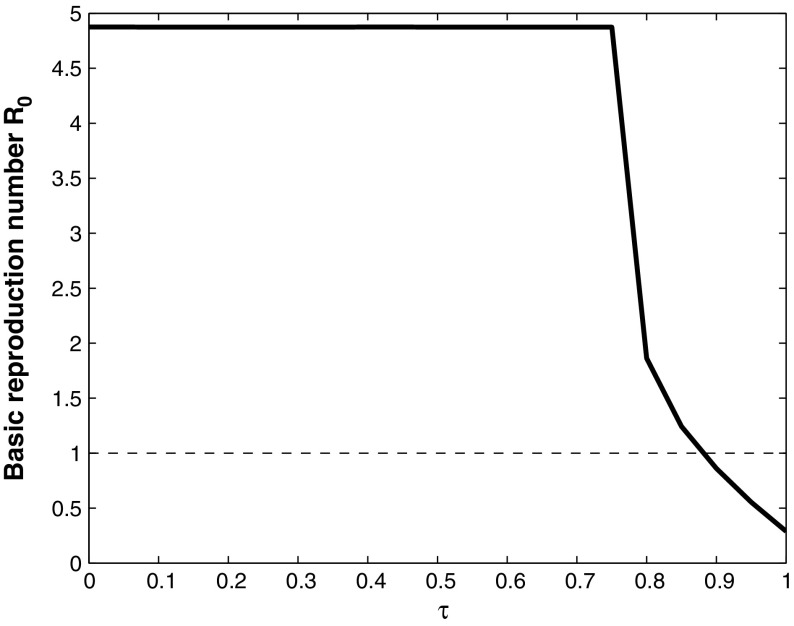
Basic reproduction number $R_0$, plotted against $\tau$, with $\beta = 0.1$, $q = 0.9$, $\gamma = 0$, $\varepsilon = 0.02$, and the other parameter values given in Table 1. If $\tau >0.9$ the basic reproduction number $R_0<1$, and WNv will die out

**Fig. 6:**
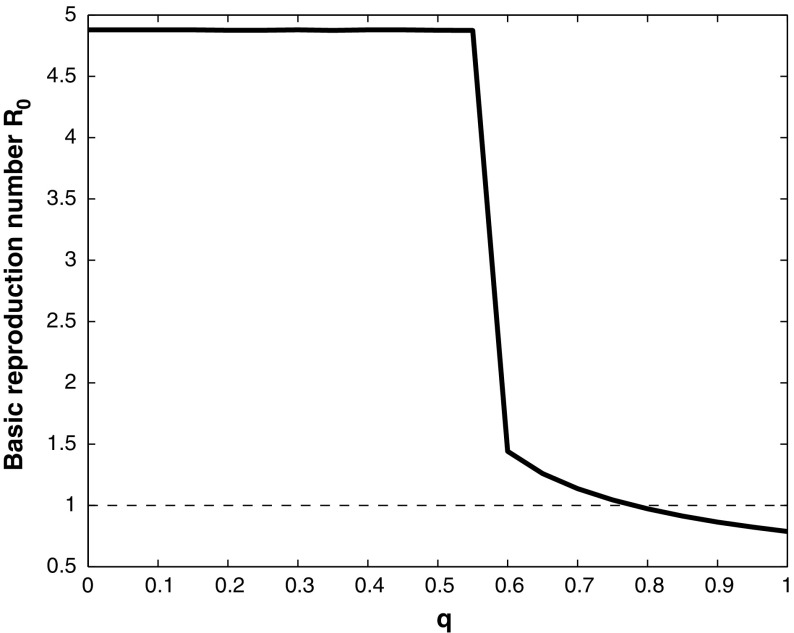
Basic reproduction number $R_0$, plotted against *q*, with $\beta = 0.1$, $\tau = 0.9$, $\gamma = 0$, $\varepsilon = 0.02$, and the other parameter values given in Table 1. If $q>0.8$ the basic reproduction number $R_0<1$, and WNv will die out

**Fig. 7:**
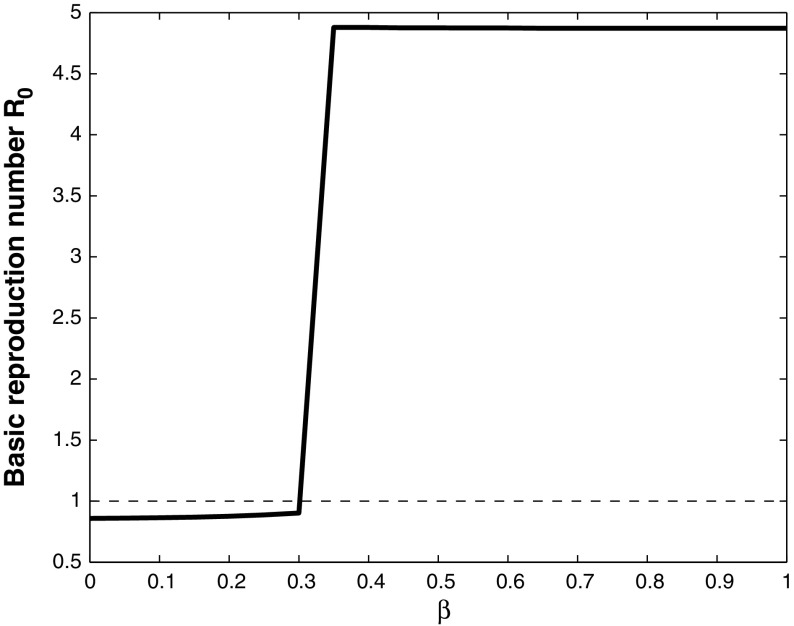
Basic reproduction number $R_0$, plotted against $\beta$, with $\tau = 0.9$, $q = 0.9$, $\gamma = 0$, $\varepsilon = 0.02$, and the other parameter values given in Table 1. If $\beta <0.3$ the basic reproduction number $R_0<1$, and WNv will die out

**Fig. 8:**
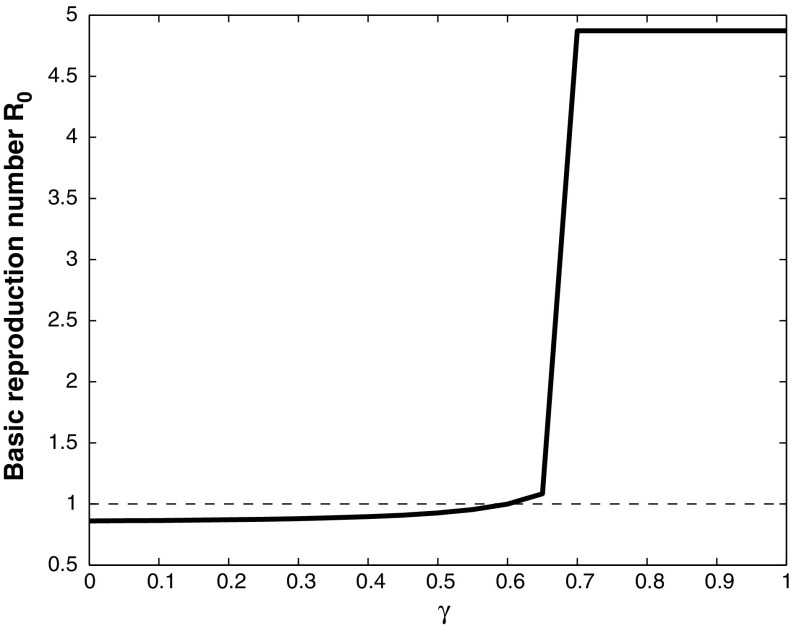
Basic reproduction number $R_0$, plotted against $\gamma$, with $\beta = 0.1$, $q = 0.9$, $\tau = 0.9$, $\varepsilon = 0.02$, and the other parameter values given in Table 1. If $\gamma <0.65$ the basic reproduction number $R_0<1$, and WNv will die out

In the simulations shown in Figs. 1–8, we set $\lambda (F_{total}):=re^{-F_{total}/k}$, where *r* is the maximum per-capita mosquito egg-laying rate, and *k* measures intra-specific competition among female mosquitoes. It should be noted that our model assumes that the mosquito population persists annually, as for example in the tropical climates of South-East Asia.

**Table 1:** Definition of parameters

Symbol	Definition	Value
$\mu _m$	Per-capita mortality rate of male mosquitoes	1/20
$\mu _f$	Per-capita mortality rate of female mosquitoes	1/20
$\mu _{wm}$	Per-capita mortality rate of *W*-infected male mosquitoes	1/20
$\mu _{wf}$	Per capita mortality rate of *W*-infected female mosquitoes	1/20
*r*	Maximum per-capita mosquito egg-laying rate	30
*k*	Competition coefficient for mosquitoes	5000
$\beta$	Fitness cost of *W*-infection on reproduction	$\in [0,1]$
$\tau$	Maternal transmission rate of *Wolbachia*	$\in [0,1]$
*q*	Strength of CI due to *W*-infection	$\in [0,1]$
$\gamma$	Male killing rate due to *W*-infection	$\in [0,1]$
$\alpha _f$	Per-capita *W*-free mosquito biting rate	0.09
$\alpha _{fw}$	Per-capita *W*-infected mosquito biting rate	0.09
$p_{bf}$	WNv transmission coefficient from birds to mosquitoes	0.16
$$p_{fb} $$	WNv transmission coefficient from mosquitoes to birds	0.88
$\mu _b$	Natural per-capita mortality rate of birds	$1/(365\times 3)$
$\mu _{bi}$	WNv-induced per-capita death rate of birds	0.1
$\Pi (B)$	Birth rate of birds	100/365
$\nu _f$	Per-capita rate at which *W*-free mosquitoes	
	complete WNv-latency and become WNv-infectious	$\in [0,1]$
$\varepsilon$	$\varepsilon \, \nu _f$ is the per-capita rate at which *W*-infected mosquitoes	
	complete WNv-latency and become WNv-infectious	
$\nu _b$	Per-capita rate at which exposed birds become infectious	0.2
$\nu _i$	Per capita rate at which infectious birds recover	0.2

Time is measured in days, and rates in day$^{-1}$. *W* stands for *Wolbachia *so that, for example, *W*-infection means *Wolbachia *infection. Parameter values have been chosen purely to demonstrate possible solution behaviour and are not based on data

## Conclusion

In this paper we have derived a detailed sex-structured model for a mosquito population infected with *Wolbachia *. The model captures many of the well-known key effects of *Wolbachia *infection, including cytoplasmic incompatibility, male killing, reduction in reproductive output and incomplete maternal transmission of the *Wolbachia *infection. Our analysis shows that the mosquito population can stabilise at a *Wolbachia *free equilibrium under certain circumstances, which include situations when inequality (2.11) holds. Such circumstances include, for example, if *Wolbachia *infection significantly reduces reproductive output, and/or *Wolbachia *infection significantly lowers female life expectancy. We also showed that if $\tau =1$, i.e. maternal transmission of *Wolbachia *is complete, then the mosquito population can stabilise at an equilibrium in which all mosquitoes are infected with *Wolbachia*. This happens in the case of sufficiently high cytoplasmic incompatibility. In the case of $\tau$ close to 1 we have shown that *Wolbachia *infected mosquitoes can coexist with small numbers of uninfected mosquitoes. We have also shown that under some additional assumptions our model has multiple coexistence steady states.

We extended the sex-structured mosquito population model (2.2)–(2.5) to include West Nile virus, which is spread by birds and mosquitoes, treating WNv as an SEI infection for mosquitoes, and as an SEIR infection for birds. We were motivated by results recently reported in Hussain (2013), which suggest that a particular strain of *Wolbachia *substantially reduces WNv replication in the mosquito species *Aedes aegypti*. We modelled this crucial phenomenon by incorporating a small parameter $\varepsilon$, the reciprocal of which is proportional to the time spent in the WNv exposed class for *Wolbachia *infected mosquitoes. This enabled us to assess the potential of *Wolbachia *infection to eradicate WNv via its effect on WNv replication in *Wolbachia *infected mosquitoes. Notably the expression we obtained for the basic reproduction number $R_0$ suggests that WNv will be eradicated if at the steady state the overwhelming majority of mosquitoes are infected with *Wolbachia*, and the *Wolbachia *infection substantially reduces WNv replication in mosquitoes. The first of these hypotheses is in fact shown to hold for a number of *Wolbachia *strains and mosquito species, see e.g. Engelstädter and Telschow (2009).
